# Applications of machine learning algorithms to detect digital addiction: a meta-analysis

**DOI:** 10.3389/fpsyt.2026.1789188

**Published:** 2026-06-23

**Authors:** Mengyang Xu, Yandie Zheng, Xingfa Long

**Affiliations:** 1 Quzhou College of Technology, Quzhou, China; 2 Quzhou University, Quzhou, China

**Keywords:** automated detection, classification accuracy, digital addiction, machine learning, meta-analysis

## Abstract

Digital addiction (DA) has emerged as a significant global concern, yet traditional diagnostic methods relying on self-report questionnaires face subjective bias and threshold inconsistencies. Recent advances in machine learning (ML) offer promising alternatives for automated DA detection. This study conducted a systematic meta-analysis of 64 eligible studies (75 independent datasets; N = 165,624), employing both single-group proportion and bivariate diagnostic test accuracy (DTA) models. The pooled classification accuracy was 0.87 (95% CI [0.85, 0.90]), and the DTA framework yielded a robust AUC of 0.92, with balanced sensitivity and specificity (both 0.86). Subgroup analyses showed high accuracy across subtypes, particularly for internet (0.90) and social media addiction (0.86). Accuracy was comparable between survey-based and physiological data, though physiological markers demonstrated superior specificity (0.90). These findings underscore the potential of ML-driven tools as scalable screening instruments while emphasizing the need for representative sampling and standardized diagnostic criteria to advance digital mental health practice.

## Introduction

As an emerging research area and umbrella term encompassing problematic use of the internet and digital technologies, digital addiction (DA) refers to a broad range of behavioral addictions stemming from human-machine interaction, typically characterized by excessive and impulsive use, withdrawal symptoms, tolerance, and conflict (1). Recent global prevalence estimates reveal that various forms of DA are increasingly common, with reported rates of 26.99% for smartphone addiction, 17.42% for social media addiction, and 14.22% for internet addiction, and different subtypes exhibit distinct behavioral patterns and clinical manifestations, which may lead to differential detection performance of machine learning (ML) models (2). This represents an increasing trend over time compared to earlier prevalence estimates (e.g., 3, 4). A growing body of research has also highlighted that DA is a significant risk factor for compromised health, academic and work performance, and diminished social functioning, often accompanied by considerable distress in personal, family, and social well-being (e.g., 5–7).

In response to increasing global concerns regarding the prevalence of DA and its adverse effects, researchers have actively pursued precise assessment instruments and technological solutions for early detection. Traditional diagnosis primarily relies on self-report questionnaires, which suffer from three critical limitations: (1) a reliance on subjective recall and self-disclosure, making them prone to response bias and social desirability effects; (2) the lack of a unified diagnostic threshold across different scales, leading to inconsistent findings; and (3) an inability to capture dynamic behavioral trajectories and physiological responses. Consequently, large-scale, objective early screening in clinical and educational settings remains a challenge (8, 9).

In recent years, adoption of artificial intelligence (AI) in addiction research has expanded significantly, allowing both researchers and clinicians to uncover more nuanced insights into addictive behaviors and associated disorders (10). By leveraging sophisticated algorithms to interpret complex datasets, AI streamlines and automates the analysis of DA patterns, effectively replacing time-consuming manual scoring methods. Ongoing research efforts are actively focused on creating automated systems capable of identifying DA using demographic information, psychological characteristics, personality, behavioral data (e.g., web browsing history, 11), and physiological signals (e.g., heart rate variability, 12).

Automated tools for detecting DA have been widely adopted and show great potential for clinical practice, yet many theoretical and methodological challenges remain unaddressed. Artificial intelligence assisted assessment serves as a practical alternative to conventional evaluation methods. Individual studies have proven that ML and deep learning (DL) algorithms work well in identifying DA, but relevant findings remain scattered without systematic quantitative synthesis, which limits their guidance for real clinical practice.

Existing studies still have two obvious methodological limitations. Researchers have not pooled overall diagnostic performance of ML models, including accuracy, sensitivity, specificity and AUC. In addition, few studies have explored the causes of high inter-study heterogeneity, as well as how diagnostic criteria, data quality control and algorithm selection affect model performance (13). Biased raw data and improper algorithm training inevitably weaken classification ability, and such methodological defects have long been overlooked in relevant research (14). Differences in algorithm selection, data sources and validation methods also lead to inconsistent research results, hindering the clinical translation of existing findings.

Given these research gaps, this study conducted a systematic meta-analysis with two core aims. It first evaluated the overall diagnostic performance of ML models in DA screening through key diagnostic indicators. It further analyzed how major factors such as diagnostic standards, algorithm types and data modalities influence model performance. This study aims to clarify the practical application of ML in DA identification, verify the clinical value of intelligent screening tools, and provide reliable evidence for future research and practical application.

## Methods

### Literature search

Our work aimed to identify studies employing ML techniques for the detection of DA. A comprehensive and systematic literature search was conducted across four major electronic databases-PubMed, Web of Science, APA PsycInfo, and Google Scholar-covering all publications from database inception through August 31, 2025. The search strategy followed PRISMA and used a broad combination of keywords related to DA and ML methodologies. Search terms encompassed digital-related terms (“internet” OR “digital” OR “screen” OR “cyber*” OR “net” OR “online” OR “media” OR “electronic device*” OR “electronic gadgets” OR “computer” OR “mobile” OR “phone” OR “smartphone” OR “television” OR “TV” OR “video” OR “facebook” OR “game” OR “gaming”), addiction-related terms (“addict*” OR “use” OR “dependen*” OR “overuse” OR “abuse” OR “disorder” OR “excessive” OR “effects” OR “habits” OR “misuse” OR “pathological” OR “problem*” OR “compulsive” OR “heavy”), ML indicators (“artificial intelligence” OR “machine learning” OR “ML” OR “DL” OR “deep learning” OR “cluster” OR “ensemble learning” OR “algorithm” OR “sentiment analysis” OR “reinforcement learning”), and diagnostic outcomes (“predict*” OR “classifi*” OR “detect*” OR “identif*” OR “extraction” OR “evaluat*” OR “diagnos*”). Full database-specific search strings and stepwise retrieval processes are detailed in **Supplementary Table 1**. As the study used only publicly available data from existing literature, ethical approval was not applicable. This study was not prospectively registered in any systematic review registry.

### Study selection

In line with PRISMA guidelines, this study established explicit inclusion and screening criteria based on the PICOS framework to guarantee scientific and rigorous literature selection. Given our mixed analytical design combining single-group proportion and diagnostic test accuracy meta-analyses, the Comparator component corresponds to the reference standard for digital addiction diagnosis. The enrolled participants included individuals of all age groups with all subtypes of digital addiction. All retrieved literatures were screened and assessed manually. Duplicated records were removed via manual comparison, and irrelevant studies were excluded, while eligible documents closely related to the research theme were retained. Potentially qualified literatures were further evaluated by full-text reading to check their compliance with preset criteria, and relevant research information was recorded in detail. Any disagreements arising during the screening process were resolved through full discussion. If consensus could not be reached, a final judgment was rendered by senior researchers with expertise in systematic reviews and meta-analysis to ensure the objectivity and credibility of the screening process.

### Data extraction

Two independent trained reviewers independently carried out data extraction by adopting a unified standardized extraction form. Prior to formal data extraction, the form was revised and optimized through pilot extraction of five randomly selected studies to standardize data extraction procedures. All extracted information was systematically sorted and compiled into a unified summary table for unified management and inspection, ensuring the whole process of data recording transparent and traceable.

The extracted information involved all preset core indicators, including the first author, sample size, relevant parameters of confusion matrix, basic data characteristics, publication status, literature category, diagnostic gold standard of DA, algorithm category and specific models, validity verification schemes, model validation approaches, data presentation forms, data leakage prevention strategies and specific subtypes of DA.

Disagreements during data extraction were resolved through in-depth discussion between the two researchers, with reference to the original literature. Unresolved discrepancies were adjudicated by a senior researcher with expertise in both DA and ML. After finishing preliminary extraction, the two researchers randomly selected ten percent of all included studies for mutual inspection against original literatures, so as to verify the accuracy and integrity of extracted data and avoid information omission and wrong entry. In line with the general research norms of machine learning-based diagnostic test accuracy meta-analysis, only the optimal performance model in each dataset was adopted for subsequent statistical analysis (13, 15–17).

### Study quality and risk of bias assessment

Two independent blinded assessors evaluated the methodological quality and risk of bias of all included studies using the Quality Assessment of Diagnostic Accuracy Studies 2 (QUADAS-2) tool (18), a standardized instrument specifically developed for diagnostic test accuracy meta-analyses. This tool includes four domains for assessing risk of bias (patient selection, index test, reference standard, and flow and timing) and three domains for evaluating applicability concerns (patient selection, index test, and reference standard), with each domain rated as low risk, high risk, or unclear risk according to standardized signaling questions. Any disagreements between the two assessors during the evaluation were resolved through thorough discussion, and a third senior reviewer was consulted to make the final judgment if consensus could not be reached.

### Meta-analytic procedure

All statistical analyses were conducted on studies assessed for methodological quality via the QUADAS-2 tool. Consistent with mainstream analytical specifications of machine learning-related diagnostic meta-analysis, only the best-performing model from each original dataset was selected for subsequent pooling analysis to eliminate interference from repeated and non-independent research results.

This study adopted two analytical approaches to comprehensively evaluate the detection performance of ML models in identifying DA. Single-group proportion meta-analysis (19) was used to synthesize the overall pooled classification accuracy. Meanwhile, diagnostic test accuracy meta-analysis (20) was performed based on two-by-two confusion matrix data to calculate pooled sensitivity, specificity and other core diagnostic indicators. Hierarchical summary receiver operating characteristic curves (21) were further constructed to intuitively display the comprehensive diagnostic efficiency of included models.

Subgroup analyses were carried out on pooled accuracy, sensitivity and specificity to explore potential sources of inter-study heterogeneity, stratified by eleven preset influencing factors including data type, publication status, literature type, diagnostic gold standard, algorithm category, specific algorithm, validity validation mode, model validation method, data modality, data leakage control measure and addiction subtype. Corresponding statistical methods were applied to test publication bias for single-group proportion meta-analysis and diagnostic test accuracy meta-analysis respectively (22, 23). All analytical procedures strictly complied with the PRISMA-DTA reporting guidelines (20).

All statistical analyses were performed in R (version 4.3.1) using the meta package for proportion meta-analysis and the mada package for diagnostic test accuracy (DTA) synthesis. For classification accuracy (a proportional outcome), the Freeman-Tukey double arcsine transformation (24) was applied to address non-normality and stabilize variance. A random-effects model based on restricted maximum likelihood (REML) (25) was used to estimate pooled effect sizes. Between-study heterogeneity was quantified using the *I*² statistic (26). For DTA synthesis, a bivariate random-effects model was used to jointly pool sensitivity and specificity while accounting for their inherent negative correlation. The HSROC model was used to calculate the area under the curve (AUC) for comprehensive diagnostic performance evaluation.

A two-sided *p* < 0.05 was considered statistically significant for all hypothesis tests, including tests for publication bias.

## Results

### Studies included in the meta-analysis

Initial literature retrieval obtained 522 records from PubMed, Web of Science, APA PsycInfo and Google Scholar, and another 18 records were supplemented via citation searching, with a total of 540 records collected. After eliminating 170 duplicate literatures, 370 records entered title and abstract screening. A total of 237 records were excluded at this stage, and the remaining 133 records were further assessed by full-text reading.

In the full-text screening stage, 69 studies were excluded for specific reasons, including reviews and theoretical papers (n = 12), studies irrelevant to DA research (n = 15), non-English publications (n = 2), studies without valid research samples (n = 5), studies lacking effect size and accuracy data (n = 26), researches focusing on craving and gaming behaviors rather than addiction symptoms (n = 3), webpage classification-related studies (n = 3), studies with overlapping research samples (n = 2), as well as literatures with incomplete data and no valid reply from authors (n = 1).

**Figure 1:**
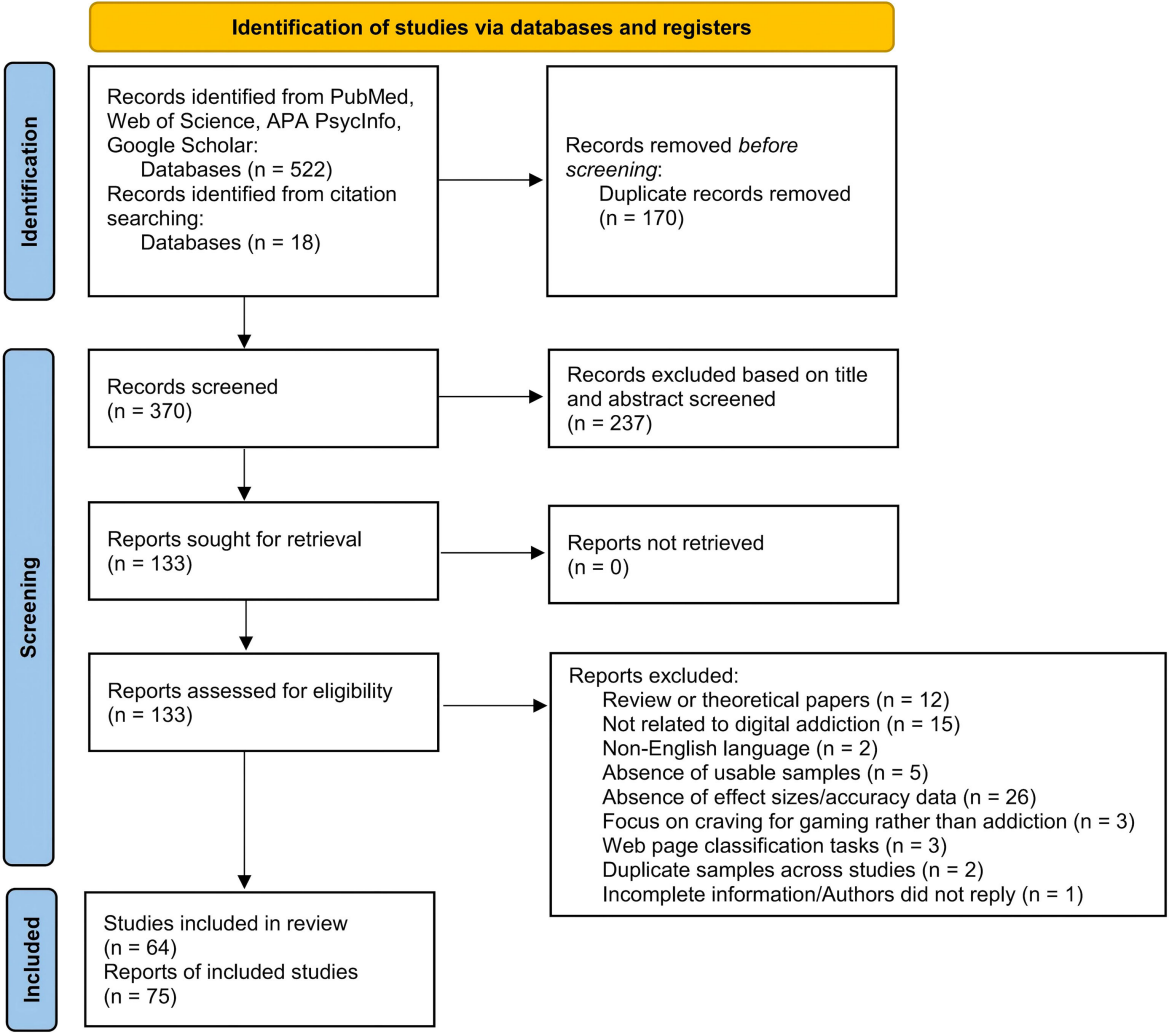
PRISMA flow diagram of the study selection process.

Finally, 64 eligible studies containing 75 independent datasets were incorporated into this meta-analysis, covering a total of 165,624 participants. The whole literature screening process is displayed in the PRISMA flow chart (**Figure 1**). The complete PRISMA 2020 Checklist (aligned with diagnostic test accuracy meta-analysis standards) is provided in **Supplementary Table 2**. Among all included datasets, 34 belonged to unpublished empirical data, including conference papers, book chapters, preprints and unpeer-reviewed dissertations, and the other 41 datasets came from peer-reviewed formal publications. The included studies were published between 2013 and 2025, with sample sizes ranging from 8 to 45,153. All included studies are marked with asterisks in **Supplementary Table 3**, and detailed bibliographic information, methodological characteristics and performance metrics for all unpublished datasets are summarized in **Supplementary Table 4**.

In terms of analytical models, 56 datasets adopted traditional ML algorithms, 16 used DL algorithms, and 3 applied ensemble classification models. Random Forest was the most commonly used model (k = 20), which was largely attributed to its stable performance in processing high-dimensional and nonlinear psychological indicators (27, 28). This meta-analysis involved six subtypes of DA, namely smartphone addiction (k = 12), gaming addiction (k = 24), internet addiction (k = 27), online shopping addiction (k = 4), pornography addiction (k = 2) and social media addiction (k = 6).

All data extracted from included studies are presented in **Supplementary Table 5**, and all relevant R analysis codes are publicly available at https://osf.io/tps6d/overview.

### Methodological quality of included studies

Following the inclusion of eligible samples, we further assessed the methodological quality and overall risk of bias across all 75 included samples using the QUADAS-2 tool.

In terms of risk of bias assessment, around 35% of samples were rated at high risk within the patient selection domain, which was mainly attributed to non-random sampling and convenience sampling designs. By contrast, over 90% of samples attained low risk of bias in both index test and flow and timing domains, indicating that most studies adopted rigorous validation procedures for ML models and strictly followed preset research protocols. Ratings for the reference standard domain were inconsistent across studies; a large number of samples were judged as high risk, primarily because diagnostic gold standards for DA were not unified, and the confirmation methods of addictive status varied greatly among different studies.

With regard to applicability concerns, several samples presented high applicability concerns in patient selection, as their recruited participants failed to fully represent general populations and clinical populations suitable for DA screening. Nearly all samples showed low applicability concerns in the index test domain, proving that the included ML models were reasonably constructed and validated for DA identification. In addition, the applicability performance of the reference standard domain differed notably, and some adopted reference standards could not well match the formal clinical definition of DA.

Detailed domain-specific QUADAS-2 bias risk evaluation outcomes and visualized summary results of all included samples are available in **Supplementary Table 6**.

### The pooled proportions of classification accuracy

**Figure 2:**
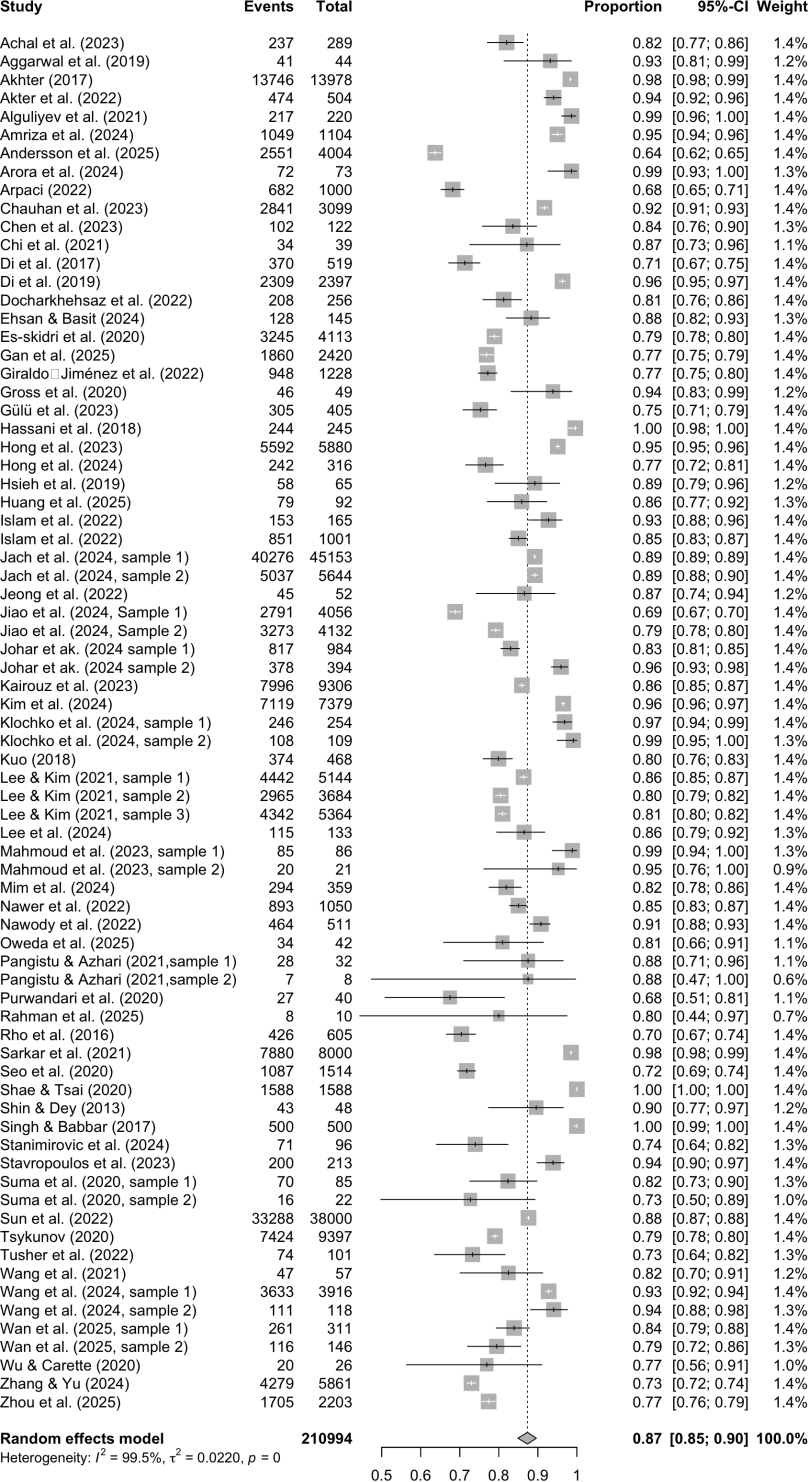
Forest plot of classification accuracy for the main random-effects models.

Forest plots were used to visually summarize the extracted results, presenting pooled classification accuracy along with associated 95% confidence intervals (CIs). As illustrated in **Figure 2**, the aggregated classification accuracy for identifying DA through ML algorithms was estimated at 0.87 (95% CI [0.85, 0.90]) under a random-effects model (19).

### Diagnostic test accuracy meta-analysis outcomes

Extracted 2×2 diagnostic data were visualized using forest plots to present pooled estimates of sensitivity and specificity with corresponding 95% confidence intervals (CIs). A bivariate random-effects meta-analysis (29) with the restricted maximum likelihood (REML) estimator was conducted to assess the diagnostic accuracy of ML models for DA. The pooled sensitivity was 0.86 (95% CI [0.81, 0.90]) and the pooled specificity was 0.86 (95% CI [0.81, 0.89]). The overall area under the HSROC curve (AUC) was 0.92, with a normalized partial AUC of 0.93.

The initial high heterogeneity (*I*² = 99.5%) for classification accuracy was substantially reduced to 20.90% in the DTA meta-analysis, where between-study heterogeneity was calculated using the Zhou and Dendukuri method (30). A weak negative correlation of −0.19 was observed between the transformed sensitivity and false positive rate parameters. Subgroup analysis stratified by data modality showed that models using physiological data yielded a higher pooled specificity of 0.90, which could effectively reduce false-positive identifications in large-scale screening scenarios.

**Figure 3:**
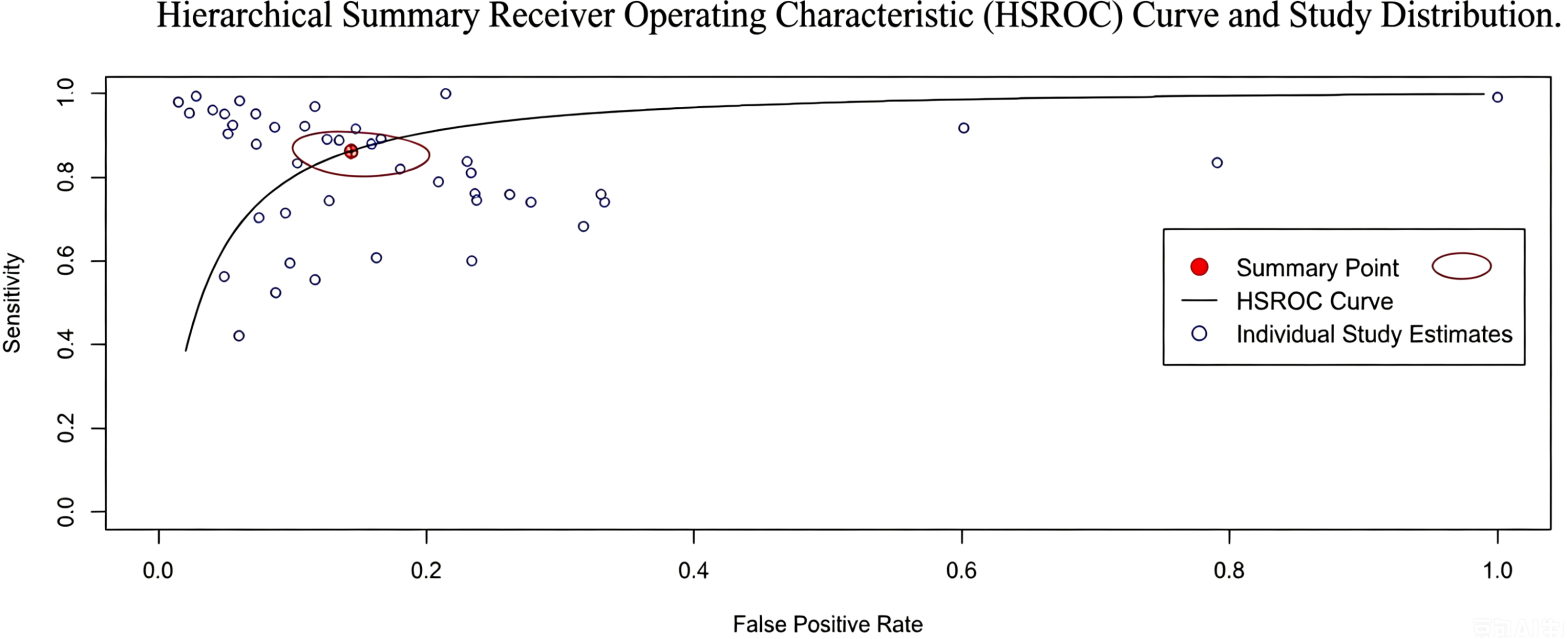
Hierarchical summary receiver operating characteristic (HSROC) curve of machine learning models for digital addiction detection.

The HSROC curve is displayed in **Figure 3**. The solid black line represents the overall diagnostic capacity across different thresholds; the red circle denotes the pooled summary point of sensitivity and specificity, surrounded by a red 95% confidence ellipse; blue open circles represent the individual estimates from each included study. The overall AUC of 0.92 indicates excellent discriminatory power of ML models for identifying DA.

### Subgroup analyses

Subgroup analyses were conducted to examine the influence of key methodological characteristics on three core diagnostic metrics: classification accuracy, sensitivity, and specificity. To ensure statistical robustness, subgroups with fewer than 3 independent samples (k < 3) were excluded from all analyses (31), and all subgroup estimates were validated prior to reporting. For classification accuracy, subgroup analyses by gold standard type revealed three distinct groups: studies using behavioral data (k = 15) yielded a pooled accuracy of 0.81 (95% CI [0.76, 0.86]), those using self-report questionnaires (k = 56) yielded 0.89 (95% CI [0.86, 0.91]), and those using clinical interviews (k = 4) yielded 0.93 (95% CI [0.84, 0.98]). Subgroup differences were statistically significant (*χ*² = 7.84, *p* = 0.02*). For sensitivity, analyses stratified by specific algorithm type showed that support vector machines (SVM, k = 15) achieved the highest pooled sensitivity of 0.92 (95% CI [0.86, 0.96]), followed by random forest (k = 11) at 0.82 (95% CI [0.72, 0.90]), and convolutional neural networks (CNN, k = 3) at 0.68 (95% CI [0.41, 0.90]). Subgroup differences were statistically significant (*χ*² = 7.86, *p* = 0.02*). For specificity, subgroup analyses by data type indicated that models using physiological data (k = 7) had the highest pooled specificity of 0.90 (95% CI [0.85, 0.94]), followed by those using unpublished data (k = 21) at 0.84 (95% CI [0.74, 0.92]), and survey-based models (k = 38) at 0.83 (95% CI [0.76, 0.89]). Subgroup differences showed a marginally significant trend (*χ*² = 3.03, *p* = 0.08^+^). Detailed subgroup results for all prespecified moderators across classification accuracy, sensitivity, and specificity are presented in **Supplementary Table 7**.

### Publication bias

**Figure 4:**
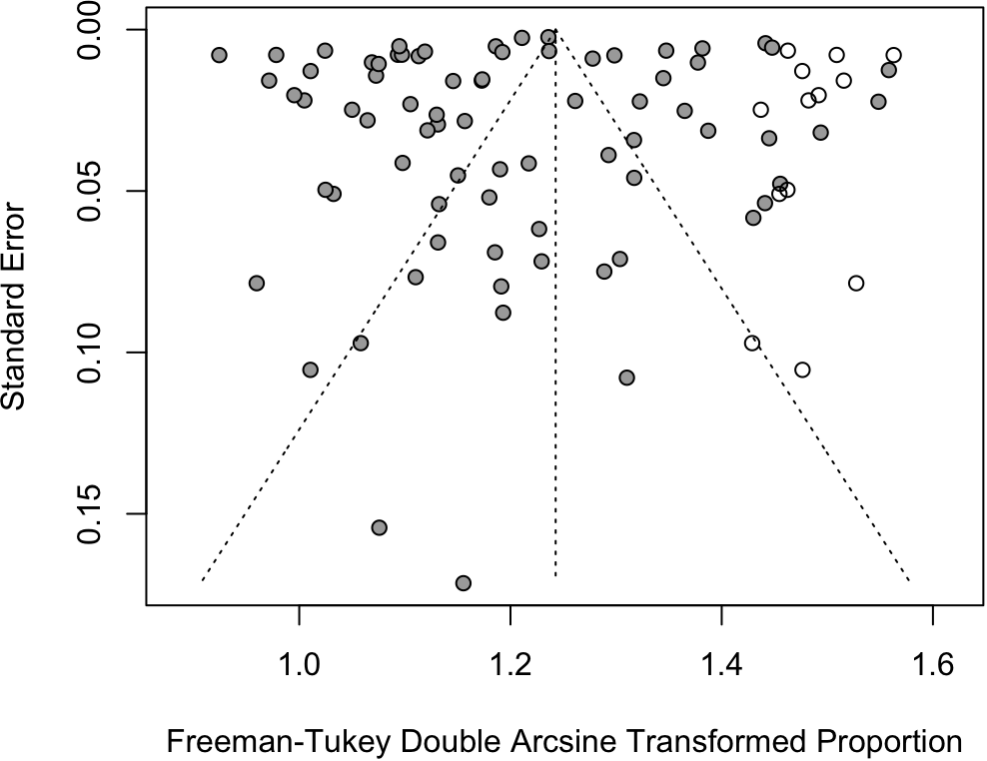
Funnel plot of classification accuracy after trim-and-fill adjustment for publication bias.

To assess potential publication bias in the meta-analysis of classification accuracy, we applied both the random-effects trim-and-fill procedure (22) and Egger’s regression test (23). First, the pooled accuracy estimate was 0.87 prior to adjustment and marginally increased to 0.90 after applying the trim-and-fill method (see **Figure 4**), reflecting a negligible absolute change (<.05). Second, Egger’s test produced non-significant results (*p* >.05), suggesting no compelling evidence of publication bias affecting the reported classification accuracy in these subgroups.

## Discussion

This systematic meta-analysis was performed to address the methodological gaps in machine learning-assisted detection of DA. Its core aims were to comprehensively assess the diagnostic performance of ML algorithms for DA using classification accuracy, sensitivity, specificity, and AUC as core metrics, and to explore how gold standard type, algorithm category, and data modality moderate model performance while accounting for the high between-study heterogeneity uncovered in our analyses. Through this synthesis, we clarify the current state of ML applications in DA detection, validate the clinical promise of AI-assisted screening tools, and offer evidence-based directions for subsequent research and practice.

This meta-analysis offers several key strengths compared to previous narrative reviews and quantitative syntheses in this field. First, it includes the largest sample size to date (N = 165,624) covering 75 independent datasets published up to August 2025, providing the most comprehensive quantitative synthesis of ML-based DA detection performance. Second, it adopts a dual-analytic approach combining single-group proportion meta-analysis and bivariate DTA meta-analysis, which addresses the limitations of relying solely on accuracy as a performance metric. Accuracy is a misleading metric in DA screening due to the low prevalence of DA in general populations (2), which inflates performance estimates for models that bias toward predicting the majority class. More importantly, accuracy fails to distinguish between false negatives (missed cases) and false positives (misdiagnoses), which have vastly different clinical consequences. The dramatic reduction in heterogeneity from 99.5% for accuracy to 20.9% in the DTA framework further confirms that much of the variability in previous findings was an artifact of overreliance on this unstable metric, rather than true differences in model performance. Third, it systematically explores 11 potential sources of between-study heterogeneity, providing critical insights into the factors influencing model performance.

Across all included studies, ML algorithms showed consistently strong diagnostic performance for DA. The pooled classification accuracy reached 0.87, reflecting reliable overall discrimination of at-risk individuals. Using the diagnostic test accuracy (DTA) framework, we further observed a high AUC of 0.92, with balanced pooled sensitivity and specificity both standing at 0.86. Compared with traditional DA assessment, which depends largely on self-report questionnaires and is limited by subjective bias and inconsistent diagnostic thresholds, machine learning-driven automated detection offers a more objective and standardized alternative. The strong diagnostic metrics obtained in this study further support its practical value for large-scale screening settings.

A central finding of this study relates to the extreme between-study heterogeneity detected in the initial accuracy analysis. Heterogeneity for classification accuracy was initially as high as 99.5% but dropped substantially to 20.9% after applying the DTA framework, indicating that much of this variability was an artifact of overreliance on a single metric rather than true differences in model performance. Follow-up moderator analyses confirmed that gold standard definition, algorithm type, and data modality together explained this heterogeneity. Studies that used structured clinical interview-based diagnostic criteria yielded a pooled accuracy of 0.93, notably higher than the 0.81 seen in studies using only behavioral data. For sensitivity, support vector machines performed best at 0.92, followed by random forests at 0.82 and convolutional neural networks (CNN) at 0.68, whereas CNN achieved the highest specificity at 0.91. Regarding data modality, physiological data produced a higher pooled specificity of 0.90, compared with 0.83 for survey-based data. These patterns confirm that variability in results stems primarily from methodological differences rather than genuine divergence in algorithm effectiveness (14).

We also observed meaningful differences in model performance across DA subtypes. After excluding subgroups with insufficient sample sizes, models for internet addiction achieved the highest accuracy at 0.90, followed by social media addiction at 0.86, gaming addiction at 0.85, and smartphone addiction at 0.84. Small sample numbers prevented formal comparisons for online shopping addiction and pornography addiction, pointing to a clear need for more research on these understudied forms of DA. Although overall accuracy did not differ significantly between survey and physiological data, the two modalities serve distinct clinical purposes: survey data are low-cost and practical for broad population screening, while physiological data deliver higher specificity that supports more confident confirmatory assessment. No single algorithm performed best across all metrics, challenging the common assumption that more complex models automatically yield better results (32). Instead, performance depends on how well algorithm choice, feature engineering, and data type align with the intended clinical use case.

Our findings have direct implications for the clinical and public health implementation of machine learning-based DA screening tools. In large-scale settings such as schools and community sites, high-sensitivity models — particularly support vector machines combined with survey data — are preferable to minimize missed cases, and their low cost and ease of use support wide deployment. For clinical confirmatory diagnosis, high-specificity models using physiological data or CNN are more appropriate to reduce false positives and avoid unnecessary interventions. Importantly, ML tools are intended to support, not replace, professional clinical judgment. The performance benchmarks established in this study can serve as empirical references for developing and refining automated DA detection systems, helping to translate research advances into evidence-based digital mental health practice.

### Limitations

While this meta-analysis provides a comprehensive synthesis of existing evidence on ML applications for DA detection, several limitations should be acknowledged.

First, although we included a substantial body of research, the distribution of samples across addiction subtypes was highly uneven, with particularly few studies focusing on subtypes such as pornography addiction and online shopping addiction. To ensure statistical reliability, we excluded these underpowered subgroups from formal comparative analyses. This prevents us from drawing definitive conclusions about their detection accuracy and limits the generalizability of our findings on algorithmic performance across all DA subtypes, highlighting the need for future research to prioritize these understudied forms.

Second, while subgroup analyses revealed no significant differences in overall accuracy between survey-based and physiological data, the types of physiological data used varied substantially. Electroencephalography, heart rate variability, and electrodermal activity each capture distinct dimensions of physiological and psychological states, serving as key indicators of mind-body interactions. However, the relatively small number of studies employing physiological measures further constrains our ability to compare the detection efficacy of different biosignals.

Third, the finding that algorithm type did not significantly influence overall accuracy may reflect the relative homogeneity in feature engineering approaches across existing studies. Future research should more systematically examine how specific feature sets, data preprocessing strategies, and model interpretability affect prediction outcomes. Additionally, very few studies have integrated theoretical frameworks from psychology or behavioral science, resulting in a weak conceptual foundation for ML predictions. Interdisciplinary research that bridges computational techniques with core psychological constructs—such as self-regulation, impulsivity, and digital literacy—could enhance both model performance and interpretability.

Fourth, the vast majority of included studies relied on internal validation rather than external validation, which may overestimate model performance in real-world settings. Our QUADAS-2 quality assessment also identified a high risk of bias in patient selection for approximately one-third of samples, further limiting the generalizability of findings to diverse populations.

Finally, most existing studies adopted cross-sectional designs and lacked longitudinal follow-up and cross-cultural comparative data. This restricts the evaluation of temporal stability and ecological validity of ML models for DA detection across diverse populations and contexts.

### Future directions

Drawing on our findings and limitations, we identify four key priorities for future research:

First, prioritize multimodal data integration. Most existing studies rely on single-modal data, yet survey and physiological data have complementary strengths and weaknesses. Combining self-reports, objective behavioral logs, and physiological signals can capitalize on these differences to enhance both the sensitivity and specificity of DA detection.

Second, conduct rigorous external validation. Nearly all models in our review used internal validation, which can overestimate performance in real-world settings. Large-scale, multicenter validation across diverse age groups, cultural backgrounds, and clinical populations is critical to establish clinical utility.

Third, ground feature engineering in psychological theory. Current approaches rely heavily on data-driven feature selection without strong theoretical foundations, limiting model interpretability and construct validity. Future work should incorporate established addiction theories—such as self-regulation and impulse control theory—to develop more clinically actionable models.

Fourth, adopt longitudinal and cross-cultural study designs. Most existing studies are cross-sectional, which cannot assess predictive validity for future addiction onset. Longitudinal cohort studies are needed to evaluate early detection capabilities, while cross-cultural comparisons will support the development of globally applicable tools.

## Conclusion

This meta-analytic synthesis indicates that ML algorithms demonstrate robust overall diagnostic performance for DA detection, with distinct performance trade-offs observed across data modalities and algorithm types: physiological data offers superior specificity, while support vector machines achieve higher sensitivity. Nevertheless, significant heterogeneity in data modalities, algorithm architectures, and validation strategies, coupled with inadequate sample representativeness and inconsistent diagnostic criteria, constrains the direct translation of these findings to routine clinical practice. Progress in this field will depend on the establishment of standardized diagnostic criteria, the development of more clinically representative datasets, the application of multimodal fusion approaches, and prospective validation in real-world clinical workflows.

## Data Availability

All R analysis codes used in this study are also publicly available at the above OSF repository: https://osf.io/tps6d/overview.

## Supplementary material

Supplemental Table 1 Database-specific search strings and stepwise retrieval process.

Supplemental Table 2 PRISMA 2020 Checklist.

Supplemental Table 3 List of all included studies.

Supplemental Table 4 Detailed information on unpublished datasets.

Supplemental Table 5 Extracted data from all included studies.

Supplemental Table 6 QUADAS-2 risk of bias and applicability assessment results.

Supplemental Table 7 Full subgroup analysis results.
