# Supplementary material for: Applications of machine learning algorithms to detect digital addiction: a meta-analysis
Source: Front Psychiatry. 2026 Jun 23;17:1789188. doi: 10.3389/fpsyt.2026.1789188 (PMC13338699; doi:10.3389/fpsyt.2026.1789188)
Supplement: Supplemental Table 1 — Database-specific search strings and stepwise retrieval process. [file Table1.docx]

**Supplemental Material A**

To ensure complete reproducibility, we have provided the detailed search strategy for every database.

Keywords: (“internet” OR “digital” OR “screen” OR “cyber*” OR “net” OR “online” OR “media” OR “electronic device*” OR “electronic﻿gadgets” OR “computer” OR “mobile” OR “phone” OR “smartphone” OR “television” OR “TV” OR “video” OR “facebook” OR “game” OR “gaming”) AND (“addict*” OR “use” OR “dependen*” OR “overuse” OR “abuse” OR “disorder” OR “excessive” OR “effects” OR “habits” OR “misuse” OR “pathological” OR “problem*” OR “compulsive” OR “heavy”) AND (“artificial intelligence” OR “machine learning” OR “ML” OR “DL” OR “deep learning” OR “cluster” OR “ensemble learning” OR “algorithm” OR “sentiment analysis” OR “reinforcement learning”) AND (“predict*” OR “classifi*” OR “detect*” OR “identif*” OR “extraction” OR “evaluat*” OR “diagnos*”)

| Databases | Strategies | Keywords | Output |
| --- | --- | --- | --- |
| Pubmed | Step 1 | “internet” OR “digital” OR “screen” OR “cyber*” OR “net” OR “online” OR “media” OR “electronic device*” OR “electronic﻿gadgets” OR “computer” OR “mobile” OR “phone” OR “smartphone” OR “television” OR “TV” OR “video” OR “facebook” OR “game” OR “gaming” | 417702 |
|  | Step 2 | “addict*” OR “use” OR “dependen*” OR “overuse” OR “abuse” OR “disorder” OR “excessive” OR “effects” OR “habits” OR “misuse” OR “pathological” OR “problem*” OR “compulsive” OR “heavy” | 2596525 |
|  | Step 3 | #2 AND #1 | 42051 |
|  | Step 4 | “artificial intelligence” OR “machine learning” OR “ML” OR “DL” OR “deep learning” OR “cluster” OR “ensemble learning” OR “algorithm” OR “sentiment analysis” OR “reinforcement learning” | 219115 |
|  | Step 5 | “predict*” OR “classifi*” OR “detect*” OR “identif*” OR “extraction” OR “evaluat*” OR “diagnos*” | 3002807 |
|  | Step 6 | #4 AND #5 | 73235 |
|  | Step 7 | #6 AND #3 | 121 |
| APA PsycInfo | Step 1 | “internet” OR “digital” OR “screen” OR “cyber*” OR “net” OR “online” OR “media” OR “electronic device*” OR “electronic﻿gadgets” OR “computer” OR “mobile” OR “phone” OR “smartphone” OR “television” OR “TV” OR “video” OR “facebook” OR “game” OR “gaming”) AND (“addict*” OR “use” OR “dependen*” OR “overuse” OR “abuse” OR “disorder” OR “excessive” OR “effects” OR “habits” OR “misuse” OR “pathological” OR “problem*” OR “compulsive” OR “heavy”) AND (“artificial intelligence” OR “machine learning” OR “ML” OR “DL” OR “deep learning” OR “cluster” OR “ensemble learning” OR “algorithm” OR “sentiment analysis” OR “reinforcement learning”) AND (“predict*” OR “classifi*” OR “detect*” OR “identif*” OR “extraction” OR “evaluat*” OR “diagnos*” | 26 |
| Web of Science | Step 1 | “internet” OR “digital” OR “screen” OR “cyber*” OR “net” OR “online” OR “media” OR “electronic device*” OR “electronic﻿gadgets” OR “computer” OR “mobile” OR “phone” OR “smartphone” OR “television” OR “TV” OR “video” OR “facebook” OR “game” OR “gaming” | 1282233 |
|  | Step 2 | “addict*” OR “use” OR “dependen*” OR “overuse” OR “abuse” OR “disorder” OR “excessive” OR “effects” OR “habits” OR “misuse” OR “pathological” OR “problem*” OR “compulsive” OR “heavy” | 3773291 |
|  | Step 3 | #2 AND #1 | 88127 |
|  | Step 4 | “artificial intelligence” OR “machine learning” OR “ML” OR “DL” OR “deep learning” OR “cluster” OR “ensemble learning” OR “algorithm” OR “sentiment analysis” OR “reinforcement learning” | 836050 |
|  | Step 5 | “predict*” OR “classifi*” OR “detect*” OR “identif*” OR “extraction” OR “evaluat*” OR “diagnos*” | 4821572 |
|  | Step 6 | #4 AND #5 | 211122 |
|  | Step 7 | #6 AND #3 | 237 |
| Google scholar | Step 1 | “internet” OR “digital” OR “screen” OR “cyber*” OR “net” OR “online” OR “media” OR “electronic device*” OR “electronic﻿gadgets” OR “computer” OR “mobile” OR “phone” OR “smartphone” OR “television” OR “TV” OR “video” OR “facebook” OR “game” OR “gaming”) AND (“addict*” OR “use” OR “dependen*” OR “overuse” OR “abuse” OR “disorder” OR “excessive” OR “effects” OR “habits” OR “misuse” OR “pathological” OR “problem*” OR “compulsive” OR “heavy”) AND (“artificial intelligence” OR “machine learning” OR “ML” OR “DL” OR “deep learning” OR “cluster” OR “ensemble learning” OR “algorithm” OR “sentiment analysis” OR “reinforcement learning”) AND (“predict*” OR “classifi*” OR “detect*” OR “identif*” OR “extraction” OR “evaluat*” OR “diagnos*” | 138 |
