# Supplementary material for: Applications of machine learning algorithms to detect digital addiction: a meta-analysis
Source: Front Psychiatry. 2026 Jun 23;17:1789188. doi: 10.3389/fpsyt.2026.1789188 (PMC13338699; doi:10.3389/fpsyt.2026.1789188)
Supplement: Supplemental Table 2 — PRISMA 2020 Checklist. [file Table2.docx]

**Supplemental Material B**

| **Section and Topic** | **Item #** | **Checklist item** | **Location where item is reported** |
| --- | --- | --- | --- |
| **TITLE** | | |  |
| Title | 1 | Identify the report as a systematic review. | Title page (title explicitly states "A Meta-analysis", a type of systematic review) |
| **ABSTRACT** | | |  |
| Abstract | 2 | See the PRISMA 2020 for Abstracts checklist. | Abstract; Supplemental Material F (PRISMA-Abstract Checklist) |
| **INTRODUCTION** | | |  |
| Rationale | 3 | Describe the rationale for the review in the context of existing knowledge. | Introduction, paragraphs 1–4 (describes DA prevalence, limitations of traditional diagnosis, and gaps in existing ML research) |
| Objectives | 4 | Provide an explicit statement of the objective(s) or question(s) the review addresses. | Introduction, last paragraph (two core aims: evaluate overall diagnostic performance and explore moderating factors) |
| **METHODS** | | |  |
| Eligibility criteria | 5 | Specify the inclusion and exclusion criteria for the review and how studies were grouped for the syntheses. | Methods, Study Selection section (PICOS framework-based criteria); Methods, Meta-analytic procedure section (subgroup grouping criteria) |
| Information sources | 6 | Specify all databases, registers, websites, organisations, reference lists and other sources searched or consulted to identify studies. Specify the date when each source was last searched or consulted. | Methods, Literature search section (PubMed, Web of Science, APA PsycInfo, Google Scholar; last searched August 31, 2025; plus manual citation searching) |
| Search strategy | 7 | Present the full search strategies for all databases, registers and websites, including any filters and limits used. | Supplemental Material A (full database-specific search strings and stepwise retrieval processes) |
| Selection process | 8 | Specify the methods used to decide whether a study met the inclusion criteria of the review, including how many reviewers screened each record and each report retrieved, whether they worked independently, and if applicable, details of automation tools used in the process. | Methods, Study Selection section (two independent reviewers; manual screening; consensus-based disagreement resolution) |
| Data collection process | 9 | Specify the methods used to collect data from reports, including how many reviewers collected data from each report, whether they worked independently, any processes for obtaining or confirming data from study investigators, and if applicable, details of automation tools used in the process. | Methods, Data Extraction section (two independent trained extractors; pilot testing; 10% cross-validation; consensus-based disagreement resolution) |
| Data items | 10a | List and define all outcomes for which data were sought. Specify whether all results that were compatible with each outcome domain in each study were sought (e.g. for all measures, time points, analyses), and if not, the methods used to decide which results to collect. | Methods, Meta-analytic procedure section (outcomes: classification accuracy, sensitivity, specificity, AUC; only the best-performing model per dataset was selected) |
|  | 10b | List and define all other variables for which data were sought (e.g. participant and intervention characteristics, funding sources). Describe any assumptions made about any missing or unclear information. | Methods, Data Extraction section (11 preset moderators: data type, publication status, literature type, diagnostic gold standard, algorithm category, specific algorithm, validity validation mode, model validation method, data modality, data leakage prevention measure, DA subtype) |
| Study risk of bias assessment | 11 | Specify the methods used to assess risk of bias in the included studies, including details of the tool(s) used, how many reviewers assessed each study and whether they worked independently, and if applicable, details of automation tools used in the process. | Methods, Study Quality and Risk of Bias Assessment section |
| Effect measures | 12 | Specify for each outcome the effect measure(s) (e.g. risk ratio, mean difference) used in the synthesis or presentation of results. | Methods, Meta-analytic procedure section (pooled proportion for accuracy; pooled sensitivity/specificity and AUC for DTA) |
| Synthesis methods | 13a | Describe the processes used to decide which studies were eligible for each synthesis (e.g. tabulating the study intervention characteristics and comparing against the planned groups for each synthesis (item #5)). | Methods, Meta-analytic procedure section (all studies meeting inclusion criteria were included in the main synthesis; subgroups were defined a priori based on the 11 moderators) |
|  | 13b | Describe any methods required to prepare the data for presentation or synthesis, such as handling of missing summary statistics, or data conversions. | Methods, Meta-analytic procedure section (Freeman-Tukey double arcsine transformation for proportion data; 2×2 confusion matrix extraction for DTA) |
|  | 13c | Describe any methods used to tabulate or visually display results of individual studies and syntheses. | Methods, Meta-analytic procedure section (forest plots for accuracy and DTA; HSROC curve; funnel plot for publication bias; tables in supplementary materials) |
|  | 13d | Describe any methods used to synthesize results and provide a rationale for the choice(s). If meta-analysis was performed, describe the model(s), method(s) to identify the presence and extent of statistical heterogeneity, and software package(s) used. | Methods, Meta-analytic procedure section (random-effects model with REML estimation; I² statistic for heterogeneity; R software: meta package for proportions, mada package for DTA) |
|  | 13e | Describe any methods used to explore possible causes of heterogeneity among study results (e.g. subgroup analysis, meta-regression). | Methods, Meta-analytic procedure section (subgroup analyses stratified by 11 preset moderators) |
|  | 13f | Describe any sensitivity analyses conducted to assess robustness of the synthesized results. | Not conducted |
| Reporting bias assessment | 14 | Describe any methods used to assess risk of bias due to missing results in a synthesis (arising from reporting biases). | Methods, Meta-analytic procedure section (Egger's regression test; trim-and-fill procedure) |
| Certainty assessment | 15 | Describe any methods used to assess certainty (or confidence) in the body of evidence for an outcome. | Not conducted (GRADE assessment not performed) |
| **RESULTS** | | |  |
| Study selection | 16a | Describe the results of the search and selection process, from the number of records identified in the search to the number of studies included in the review, ideally using a flow diagram. | Results, Studies included in the meta-analysis section; Figure 1 (PRISMA flow diagram) |
|  | 16b | Cite studies that might appear to meet the inclusion criteria, but which were excluded, and explain why they were excluded. | Results, Studies included in the meta-analysis section (lists 9 specific reasons for excluding 69 full-text articles) |
| Study characteristics | 17 | Cite each included study and present its characteristics. | Results, Studies included in the meta-analysis section; Supplemental Material B (list of included studies); Supplemental Material C (detailed study characteristics table) |
| Risk of bias in studies | 18 | Present assessments of risk of bias for each included study. | Results, Methodological Quality of Included Studies section; Supplemental Material D (study-specific QUADAS-2 assessment matrix) |
| Results of individual studies | 19 | For all outcomes, present, for each study: (a) summary statistics for each group (where appropriate) and (b) an effect estimate and its precision (e.g. confidence/credible interval), ideally using structured tables or plots. | Results, The pooled proportions of classification accuracy section (Figure 2: accuracy forest plot); Results, Diagnostic Test Accuracy section (sensitivity/specificity forest plots); Supplemental Material C (individual study results) |
| Results of syntheses | 20a | For each synthesis, briefly summarise the characteristics and risk of bias among contributing studies. | Results, Methodological Quality of Included Studies section (summary of QUADAS-2 findings across 75 samples) |
|  | 20b | Present results of all statistical syntheses conducted. If meta-analysis was done, present for each the summary estimate and its precision (e.g. confidence/credible interval) and measures of statistical heterogeneity. If comparing groups, describe the direction of the effect. | Results, The pooled proportions section (pooled accuracy = 0.87, 95% CI [0.85, 0.90], I² = 99.5%); Results, DTA section (pooled sensitivity = 0.86, specificity = 0.86, AUC = 0.92, I² = 20.9%) |
|  | 20c | Present results of all investigations of possible causes of heterogeneity among study results. | Results, Subgroup Analyses section; Supplemental Material E (complete subgroup analysis results for all 11 moderators) |
|  | 20d | Present results of all sensitivity analyses conducted to assess the robustness of the synthesized results. | Not conducted |
| Reporting biases | 21 | Present assessments of risk of bias due to missing results (arising from reporting biases) for each synthesis assessed. | Results, Publication bias section (Egger's test p > 0.05; trim-and-fill adjustment changed accuracy from 0.87 to 0.90) |
| Certainty of evidence | 22 | Present assessments of certainty (or confidence) in the body of evidence for each outcome assessed. | Not conducted |
| **DISCUSSION** | | |  |
| Discussion | 23a | Provide a general interpretation of the results in the context of other evidence. | Discussion, paragraphs 2–4 (interprets core findings in relation to prior systematic reviews) |
|  | 23b | Discuss any limitations of the evidence included in the review. | Discussion, Limitations section (5 key limitations: uneven subtype distribution, limited physiological data, homogeneous feature engineering, lack of external validation, cross-sectional design) |
|  | 23c | Discuss any limitations of the review processes used. | Discussion, Limitations section (acknowledges exclusion of non-English studies and potential publication bias despite assessment) |
|  | 23d | Discuss implications of the results for practice, policy, and future research. | Discussion, clinical implications paragraph; Discussion, Future Directions section (4 key priorities for future research) |
| **OTHER INFORMATION** | | |  |
| Registration and protocol | 24a | Provide registration information for the review, including register name and registration number, or state that the review was not registered. | Methods, Literature search section ("This study was not prospectively registered in any systematic review registry") |
|  | 24b | Indicate where the review protocol can be accessed, or state that a protocol was not prepared. | Not prepared |
|  | 24c | Describe and explain any amendments to information provided at registration or in the protocol. | Not applicable (no registration/protocol) |
| Support | 25 | Describe sources of financial or non-financial support for the review, and the role of the funders or sponsors in the review. | Not reported |
| Competing interests | 26 | Declare any competing interests of review authors. | Not reported |
| Availability of data, code and other materials | 27 | Report which of the following are publicly available and where they can be found: template data collection forms; data extracted from included studies; data used for all analyses; analytic code; any other materials used in the review. | Results, Studies included in the meta-analysis section ("All data extracted from included studies are presented in Supplemental Material C, and all relevant R analysis codes are publicly available at [https://osf.io/tps6d/overview](https://osf.io/tps6d/overview)") |

*From:*  Page MJ, McKenzie JE, Bossuyt PM, Boutron I, Hoffmann TC, Mulrow CD, et al. The PRISMA 2020 statement: an updated guideline for reporting systematic reviews. BMJ 2021;372:n71. doi: 10.1136/bmj.n71. This work is licensed under CC BY 4.0. To view a copy of this license, visit <https://creativecommons.org/licenses/by/4.0/>

| **Section and Topic** | **Item #** | **Checklist item** | **Reported (Yes/No)** |
| --- | --- | --- | --- |
| **TITLE** | | |  |
| Title | 1 | Identify the report as a systematic review. | Yes |
| **BACKGROUND** | | |  |
| Objectives | 2 | Provide an explicit statement of the main objective(s) or question(s) the review addresses. | Yes |
| **METHODS** | | |  |
| Eligibility criteria | 3 | Specify the inclusion and exclusion criteria for the review. | No |
| Information sources | 4 | Specify the information sources (e.g. databases, registers) used to identify studies and the date when each was last searched. | No |
| Risk of bias | 5 | Specify the methods used to assess risk of bias in the included studies. | No |
| Synthesis of results | 6 | Specify the methods used to present and synthesise results. | Yes |
| **RESULTS** | | |  |
| Included studies | 7 | Give the total number of included studies and participants and summarise relevant characteristics of studies. | Yes |
| Synthesis of results | 8 | Present results for main outcomes, preferably indicating the number of included studies and participants for each. If meta-analysis was done, report the summary estimate and confidence/credible interval. If comparing groups, indicate the direction of the effect (i.e. which group is favoured). | Yes |
| **DISCUSSION** | | |  |
| Limitations of evidence | 9 | Provide a brief summary of the limitations of the evidence included in the review (e.g. study risk of bias, inconsistency and imprecision). | No |
| Interpretation | 10 | Provide a general interpretation of the results and important implications. | Yes |
| **OTHER** | | |  |
| Funding | 11 | Specify the primary source of funding for the review. | No |
| Registration | 12 | Provide the register name and registration number. | No |

*From:*  Page MJ, McKenzie JE, Bossuyt PM, Boutron I, Hoffmann TC, Mulrow CD, et al. The PRISMA 2020 statement: an updated guideline for reporting systematic reviews. BMJ 2021;372:n71. doi: 10.1136/bmj.n71. This work is licensed under CC BY 4.0. To view a copy of this license, visit <https://creativecommons.org/licenses/by/4.0/>
