# Supplementary material for: Applications of machine learning algorithms to detect digital addiction: a meta-analysis
Source: Front Psychiatry. 2026 Jun 23;17:1789188. doi: 10.3389/fpsyt.2026.1789188 (PMC13338699; doi:10.3389/fpsyt.2026.1789188)
Supplement: Supplemental Table 3 — List of all included studies. [file Table3.docx]

**Supplemental Material C**

**﻿References marked with an asterisk indicate studies included in the meta‑analysis**

^*^Achal, F. T., Ahmmed, M. S., & Aurpa, T. T. (2023). Severity detection of problematic smartphone usage (PSU) and its effect on human lifestyle using machine learning. In *2023 IEEE 8th International Conference for Convergence in Technology (I2CT)* (pp. 1-6). IEEE.

^*^Aggarwal, S., Saluja, S., Gambhir, V., Gupta, S., & Satia, S. P. S. (2020). Predicting likelihood of psychological disorders in Player Unknown’s Battlegrounds (PUBG) players from Asian countries using supervised machine learning. *Addictive Behaviors*, *101*, 106132.

^*^Akhter, S. (2018). Using machine learning to predict potential online gambling addicts (unpublished Master’s thesis). Aalto University, Espoo.

^*^Akter, M., Ritu, K. F., Habib, M. T., Rahman, M. S., & Ahmed, F. (2022). A machine learning approach to predict social media addiction during COVID-19 pandemic. In *2022 International Conference on Applied Artificial Intelligence and Computing (ICAAIC)* (pp. 401-405). IEEE.

^*^Alguliyev, R. M., Abdullayeva, F. J., & Ojagverdiyeva, S. S. (2021). Log-file analysis to identify internet-addiction in children. *International Journal of Modern Education and Computer Science*, *10*(5), 23-31.

^*^Amriza, R. N. S., & Fadhilla, C. A. (2024). Unraveling game addiction behavior among digital native students: Leveraging machine learning for insights. In *2024 IEEE International Conference on Communication, Networks and Satellite (COMNETSAT)* (pp. 437-444). IEEE.

^*^Andersson, S., Carlbring, P., Lyon, K., Bermell, M., & Lindner, P. (2025). Insights into the temporal dynamics of identifying problem gambling on an online casino: A machine learning study on routinely collected individual account data. *Journal of Behavioral Addictions*, *14*(1), 490-500.

^*^Arora, A., Chakraborty, P., & Bhatia, M. P. S. (2023). Optimizing smartphone addiction questionnaires with smartphone application and soft computing: An intelligent smartphone usage behavior assessment model. In *Soft Computing Techniques in Connected Healthcare Systems* (pp. 17-33). CRC Press.

^*^Arpaci, I. (2023). Predicting problematic smartphone use based on early maladaptive schemas by using machine learning classification algorithms. *Journal of Rational-Emotive & Cognitive-Behavior Therapy*, *41*(3), 634-643.

^*^Chauhan, S., Mittal, M., Singh, H., Kumar, S., Goel, P., & Gupta, S. (2023). Predictive analysis on student's mental health towards online mobile games using machine learning. In *2023 IEEE 15th International Conference on Computational Intelligence and Communication Networks (CICN)* (pp. 321-324). IEEE.

^*^Chen, J., Xie, Z. Y., Chen, T. Z., Lu, J. X., Zeng, N. N., & Zheng, H. (2023). Impaired reinforcement learning and behavioral activation/inhibition systems in internet addiction. Retrieved from https://assets-eu.researchsquare.com/files/rs-3272564/v1/5e4ac3fe-19b7-4c76-bfe3-9e01c75782b1.pdf?c=1711518782.

^*^Chi, H. M., Chen, L. Y., & Hsiao, T. C. (2021). Extraction of psychological symptoms and instantaneous respiratory frequency as indicators of internet addiction using rule-based machine learning. *Advances in Science, Technology and Engineering Systems Journal*, *6*(5), 203-212.

^*^Di, Z., Gong, X., Shi, J., Ahmed, H. O., & Nandi, A. K. (2017). Detection of IAD based on personality questionnaires of Chinese college students and SVMs. In *2017 10th International Congress on Image and Signal Processing, BioMedical Engineering and Informatics (CISP-BMEI)* (pp. 1-6). IEEE.

^*^Di, Z., Gong, X., Shi, J., Ahmed, H. O., & Nandi, A. K. (2019). Internet addiction disorder detection of Chinese college students using several personality questionnaire data and support vector machine. *Addictive Behaviors Reports*, *10*, 100200.

^*^Docharkhehsaz, M., Hashemi Nosratabad, T., Beirami, M., & Sattari, M. T. (2022). Investigation of the differential power of young’s internet addiction questionnaire using the decision stump tree. *Computational Intelligence and Neuroscience*, *2022*(1), 3930273.

^*^Ehsan, T., & Basit, J. (2024). Machine learning for detecting social media addiction patterns: Analyzing user behavior and mental health data. *International Journal of Innovations in Science and Technology,* *6*(4), 1789-1807.

^*^Es-Skidri, R. (2019). Gambling addiction, a machine learning approach (unpublished Master’s thesis). Politecnico di Milano, Milano.

^*^Gan, Y., Kuang, L., Xu, X. M., Ai, M., He, J. L., Wang, W., ... & Zhang, Q. (2025). Application of machine learning in predicting adolescent internet behavioral addiction. *Frontiers in Psychiatry*, *15*, 1521051.

^*^Giraldo-Jiménez, C. F., Gaviria-Chavarro, J., Sarria-Paja, M., Bermeo Varón, L. A., Villarejo-Mayor, J. J., & Rodacki, A. L. F. (2022). Smartphones dependency risk analysis using machine-learning predictive models. *Scientific Reports*, *12*(1), 22649.

^*^Gross, J., Baumgartl, H., & Buettner, R. (2020). A novel machine learning approach for high-performance diagnosis of premature internet addiction using the unfolded EEG Spectra. In *AMCIS 2020 Proceedings: 25th Americas Conference on Information Systems*. (pp. 1-8). IEEE.

^*^Gülü, M., Yagin, F. H., Gocer, I., Yapici, H., Ayyildiz, E., Clemente, F. M., ... & Nobari, H. (2023). Exploring obesity, physical activity, and digital game addiction levels among adolescents: A study on machine learning-based prediction of digital game addiction. *Frontiers in Psychology*, *14*, 1097145.

^*^Hassani, Z., Pakzad, A., & Asghari, A. (2018). An artificial predictive modeling framework for automatically detecting problematic use of internet. *International Journal of Computer Applications*, *179*(41), 31-38.

^*^Hong, Y., Rong, X., & Liu, W. (2024). Construction of influencing factor segmentation and intelligent prediction model of college students' cell phone addiction model based on machine learning algorithm. *Heliyon, 10*, e29245.

^*^Hong, S. J., Lee, D., Park, J., Kim, T., Jung, Y. C., Shon, Y. M., & Kim, I. Y. (2023). Severity identification for internet gaming disorder using heart rate variability reactivity for gaming cues: A deep learning approach. *Frontiers in Psychiatry*, *14*, 1231045.

^*^Hsieh, W. H., Shih, D. H., Shih, P. Y., & Lin, S. B. (2019). An ensemble classifier with case-based reasoning system for identifying internet addiction. *International Journal of Environmental Research and Public Health*, *16*(7), 1233.

^*^Huang, H. W., Li, P. Y., Chen, M. C., Chang, Y. X., Liu, C. L., Chen, P. W., ... & Wu, S. C. (2025). Classification of internet addiction using machine learning on electroencephalography synchronization and functional connectivity. *Psychological Medicine*, *55*, e148.

^*^Islam, M. Z., Jannat, Z., Habib, M. T., Rahman, M. S., & Islam, G. Z. (2022). Detection of Facebook addiction using machine learning. In *International Conference on Image Processing and Capsule Networks* (pp. 625-638). Cham: Springer International Publishing.

^*^Islam, S., Tusher, A. N., Mia, M. S., & Rahman, M. S. (2022,). A machine learning based approach to predict online gaming addiction in the context of Bangladesh. In *2022 13th International Conference on Computing Communication and Networking Technologies (ICCCNT)* (pp. 1-7). IEEE.

^*^Jach, T., Probierz, B., Kozak, J., Stefański, P., Dziczkowski, G., Hrabia, A., ... & Joniec, N. (2024). Identification of users in a gambling problem with the use of machine learning. In *Asian Conference on Intelligent Information and Database Systems* (pp. 263-274). Singapore: Springer Nature Singapore.

^*^Jeong, B., Lee, J., Kim, H., Gwak, S., Kim, Y. K., Yoo, S. Y., ... & Choi, J. S. (2022). Multiple-kernel support vector machine for predicting internet gaming disorder using multimodal fusion of PET, EEG, and clinical features. *Frontiers in Neuroscience*, *16*, 856510.

^*^Jiao, Y., Wong-Padoongpatt, G., & Yang, M. (2024). Detection of problem gambling with less features using machine learning methods. *arXiv preprint arXiv:2403.15962*.

^*^Johar, G., & Patel, R. (2024). A machine learning model for predicting internet addiction in teenage and young students. In *2024 IEEE 3rd World Conference on Applied Intelligence and Computing (AIC)* (pp. 285-291). IEEE.

^*^Kairouz, S., Costes, J. M., Murch, W. S., Doray-Demers, P., Carrier, C., & Eroukmanoff, V. (2023). Enabling new strategies to prevent problematic online gambling: A machine learning approach for identifying at-risk online gamblers in France. *International Gambling Studies*, *23*(3), 471-490.

^*^Kim, K., Yoon, Y., & Shin, S. (2024). Explainable prediction of problematic smartphone use among South Korea's children and adolescents using a machine learning approach. *International Journal of Medical Informatics*, *186*, 105441.

^*^Klochko, O. V., Fedorets, V. M., & Klochko, V. I. (2024). Empirical comparison of clustering and classification methods for detecting Internet addiction. *CTE Workshop Proceedings*, *11,* 273-302.

^*^Kuo, F. Y. (2024). Online social network data-driven early detection on short-form video addiction. *arXiv preprint arXiv:2407.18277*.

^*^Lee, J., & Kim, W. (2021). Prediction of problematic smartphone use: A machine learning approach. *International Journal of Environmental Research and Public Health*, *18*(12), 6458.

^*^Lee, J. Y., Song, M. S., Yoo, S. Y., Jang, J. H., Lee, D., Jung, Y. C., ... & Choi, J. S. (2024). Multimodal-based machine learning approach to classify features of internet gaming disorder and alcohol use disorder: A sensor-level and source-level resting-state electroencephalography activity and neuropsychological study. *Comprehensive Psychiatry*, *130*, 152460.

^*^Mahmoud, A., Mohamed, Y., Anter, A. M., & Zaky, A. B. (2023, December). EEG-based detection of pornography addiction: Deep learning models with attention mechanism for unveiling neurocognitive patterns. In *2023 11th International Japan-Africa Conference on Electronics, Communications, and Computations (JAC-ECC)* (pp. 73-78). IEEE.

^*^Mim, M. N., Firoz, M., Islam, M. M., Hasan, M., & Habib, M. T. (2024). A study on social media addiction analysis on the people of Bangladesh using machine learning algorithms. *Bulletin of Electrical Engineering and Informatics*, *13*(5), 3493-3502.

^*^Nawer, N., Jahan, N., Fuwad, M. M., Bhuiyan, M. H., & Kabir, I. (2022). Machine learning-based approach on predicting online shopping addiction using EEG signals (unpublished Doctoral dissertation). Brac University, Dhaka.

^*^Nawodya, A. G., & Kumara, B. T. G. S. (2022). Machine learning approach to detect online shopping addiction. In *2022 2nd international conference on advanced research in computing (ICARC)* (pp. 78-83). IEEE.

^*^Oweda, J., Schmitgen, M. M., Henemann, G. M., Gerdes, M., & Wolf, R. C. (2025). Machine learning based classification of excessive smartphone users via neuronal cue reactivity. *Psychiatry Research: Neuroimaging*, *346*, 111903.

^*^Pangistu, L. A. M., & Azhari, A. (2021). Deep learning on game addiction detection based on electroencephalogram. *Journal Media Informatika Budidarma*, *5*(3), 963-970.

^*^Purwandari, B., Wibawa, W. S., Fitriah, N., Christia, M., & Bintari, D. R. (2020). Internet addiction and mental health prediction using ensemble learning based on web browsing history. In *Proceedings of the 3rd International Conference on Software Engineering and Information Management* (pp. 155-159).

^*^Rahman, M., Mahi, A. M., Sultana, S., Churpek, M. M., & Alam, M. A. U. (2025). Detection of short-form video addiction with wearable sensors via temporally-coherent domain adaptation. In *AMIA Annual Symposium Proceedings* (p. 940-949).

^*^Rho, M. J., Jeong, J. E., Chun, J. W., Cho, H., Jung, D. J., Choi, I. Y., & Kim, D. J. (2016). Predictors and patterns of problematic Internet game use using a decision tree model. *Journal of Behavioral Addictions*, *5*(3), 500-509.

^*^Sarkar, S., Bhandary, S., & Arya, A. (2021). Effectuating supervised machine learning techniques for multiclass classification of problematic Internet and mobile usage. In *2021 International Conference on Computing, Communication, and Intelligent Systems (ICCCIS)* (pp. 1-8). IEEE.

^*^Seo, W., Kim, N., Lee, S. K., & Park, S. M. (2020). Machine learning-based analysis of adolescent gambling factors. *Journal of Behavioral Addictions*, *9*(3), 734-743.

^*^Shae, Z. Y., & Tsai, J. J. (2020, October). Deep learning mechanism for pervasive internet addiction prediction. In *2020 IEEE Second International Conference on Cognitive Machine Intelligence (CogMI)* (pp. 1-7). IEEE.

^*^Shin, C., & Dey, A. K. (2013, September). Automatically detecting problematic use of smartphones. In *Proceedings of the 2013 ACM international joint conference on Pervasive and ubiquitous computing* (pp. 335-344).

^*^Singh, A., & Babbar, S. (2017). Detecting internet addiction disorder using Bayesian networks. In *International Conference on Recent Developments in Science, Engineering and Technology* (pp. 80-95). Singapore: Springer Singapore.

^*^Suma, S. N., Nataraja, P., & Sharma, M. K. (2019, May). Internet addiction predictor: Applying machine learning in psychology. In *International Conference on Artificial Intelligence and Data Engineering* (pp. 471-481). Singapore: Springer Nature Singapore.

^*^Stanimirovic, A. S., Nikolic, M. S., Jovic, J. J., Ristic, D. I. I., Corac, A. M., Stoimenov, L. V., & Peric, Z. H. (2024). Applying eXplainable AI techniques to interpret machine learning predictive models for the analysis of problematic internet use among adolescents. *Elektronika ir Elektrotechnika*, *30*(2), 63-72.

^*^Stavropoulos, V., Zarate, D., Prokofieva, M., Van de Berg, N., Karimi, L., Gorman Alesi, A., ... & Griffiths, M. D. (2023). Deep learning (s) in gaming disorder through the user-avatar bond: A longitudinal study using machine learning. *Journal of Behavioral Addictions*, *12*(4), 878-894.

^*^Sun, S., Yang, J., Chen, Y. H., Miao, J., & Sawan, M. (2022). EEG signals based internet addiction diagnosis using convolutional neural networks. *Applied Sciences*, *12*(13), 6297.

^*^Tsykunov, N. (2020). Early detection of online gambling addiction (unpublished Master’s thesis). Tallinn University of Technology, Tallinn.

^*^Tusher, A. N., Islam, S., Islam, M. T., Sammy, M. S. R., Rahman, M. S., & Sadik, M. S. (2022). User perspective Bangla sentiment analysis for online gaming addiction using machine learning. In *2022 Sixth International Conference on I-SMAC (IoT in Social, Mobile, Analytics and Cloud)(I-SMAC)* (pp. 538-543). IEEE.

^*^Wan, X., Zeng, J., & Zhang, L. (2025). Predicting online shopping addiction: A decision tree model analysis. *Frontiers in Psychology*, *15*, 1462376.

^*^Wang, Y., Qin, Y., Li, H., Yao, D., Sun, B., Gong, J., ... & Zhu, T. (2021). Identifying internet addiction and evaluating the efficacy of treatment based on functional connectivity density: A machine learning study. *Frontiers in Neuroscience*, *15*, 665578.

^*^Wang, X., Zhang, E., Cui, Y., Huang, J., & Cheng, M. (2024). Predicting internet addiction in college students using a 1D-CNN model: Analysis of influencing factors. *DYNA: revista de la Facultad de Minas. Universidad Nacional de Colombia. Sede Medellín*, *91*(233), 66-74.

^*^Wu, Q., & Carette, J. (2020). Can deep learning predict problematic gaming?. In *2020 IEEE Conference on Games (CoG)* (pp. 662-665). IEEE.

^*^Zhang, S., & Yu, H. (2024). Online addiction analysis and identification of students by applying gd-LSTM algorithm to educational behaviour data. *Journal of Intelligent Systems*, *33*(1), 20230102.

^*^Zhou, Y., Pei, C., Yin, H., Zhu, R., Yan, N., Wang, L., ... & Huo, L. (2025). Predictors of smartphone addiction in adolescents with depression: Combing the machine learning and moderated mediation model approach. *Behaviour Research and Therapy*, *189*, 104749.
