# Supplementary material for: Applications of machine learning algorithms to detect digital addiction: a meta-analysis
Source: Front Psychiatry. 2026 Jun 23;17:1789188. doi: 10.3389/fpsyt.2026.1789188 (PMC13338699; doi:10.3389/fpsyt.2026.1789188)
Supplement: Supplemental Table 6 — QUADAS-2 risk of bias and applicability assessment results. [file Table6.docx]

**Supplementary Material F**

﻿QUADAS-2 risk of bias assessment

| Study | ﻿Risk of bias | | | |  | ﻿Applicability concerns | | |
| --- | --- | --- | --- | --- | --- | --- | --- | --- |
|  | ﻿Patient selection | ﻿Index test | ﻿Reference standard | ﻿Flow and timing |  | ﻿Patient selection | ﻿Index test | ﻿Reference standard |
| Achal et al. (2023) | H | L | H | L |  | U | L | H |
| Aggarwal et al. (2019) | H | U | L | L |  | H | L | L |
| Akhter (2017) | H | L | U | L |  | L | L | L |
| Akter et al. (2022) | H | U | L | L |  | L | L | L |
| Alguliyev et al. (2021) | U | L | U | L |  | L | L | U |
| Amriza et al. (2024) | H | L | L | L |  | U | L | L |
| Andersson et al. (2025) | L | L | H | L |  | L | L | L |
| Arora et al. (2024) | H | L | U | L |  | H | L | L |
| Arpaci (2022) | U | L | L | L |  | H | L | L |
| Chauhan et al. (2023) | L | L | H | L |  | L | L | L |
| Chen et al. (2023) | L | L | L | L |  | L | L | L |
| Chi et al. (2021) | L | L | L | L |  | L | L | L |
| Di et al. (2017) | H | L | L | L |  | L | L | L |
| Di et al. (2019) | L | L | L | L |  | L | L | L |
| Docharkhehsaz et al. (2022) | L | L | L | L |  | L | L | L |
| Ehsan & Basit (2024) | H | L | U | L |  | H | L | U |
| Es-skidri et al. (2020) | L | L | L | L |  | L | L | L |
| Gan et al. (2025) | L | L | L | L |  | L | L | L |
| Giraldo‑Jiménez et al. (2022) | L | L | L | L |  | L | L | L |

﻿ QUADAS-2 risk of bias assessment

| Study | ﻿Risk of bias | | | |  | ﻿Applicability concerns | | |
| --- | --- | --- | --- | --- | --- | --- | --- | --- |
|  | ﻿Patient selection | ﻿Index test | ﻿Reference standard | ﻿Flow and timing |  | ﻿Patient selection | ﻿Index test | ﻿Reference standard |
| Gross et al. (2020) | U | L | L | L |  | L | L | L |
| Gülü et al. (2023) | L | L | H | L |  | L | L | L |
| Hassani et al. (2018) | U | L | H | L |  | H | L | H |
| Hong et al. (2023) | U | L | L | L |  | U | L | L |
| Hong et al. (2024) | L | L | L | L |  | L | L | L |
| Hsieh et al. (2019) | H | L | L | U |  | L | L | L |
| Huang et al. (2025) | L | L | L | L |  | L | L | L |
| Islam et al. (2022) | H | L | L | L |  | L | L | L |
| Islam et al. (2022) | U | L | H | L |  | U | L | H |
| Jach et al. (2024, sample 1) | L | L | L | L |  | L | L | L |
| Jach et al. (2024, sample 2) | L | L | L | L |  | L | L | L |
| Jeong et al. (2022) | L | L | L | L |  | L | L | L |
| Jiao et al. (2024, Sample 1) | L | L | L | L |  | L | L | L |
| Jiao et al. (2024, Sample 2) | L | L | L | L |  | L | L | L |
| Johar et ak. (2024 sample 1) | U | L | L | L |  | L | L | L |
| Johar et ak. (2024 sample 2) | U | L | L | L |  | L | L | L |
| Kairouz et al. (2023) | L | L | L | L |  | L | L | L |
| Kim et al. (2024) | L | L | L | L |  | L | L | L |
| Klochko et al. (2024, sample 1) | H | L | L | L |  | H | L | L |
| Klochko et al. (2024, sample 2) | H | L | L | L |  | H | L | L |

QUADAS-2 risk of bias assessment

| Study | ﻿Risk of bias | | | |  | ﻿Applicability concerns | | |
| --- | --- | --- | --- | --- | --- | --- | --- | --- |
|  | ﻿Patient selection | ﻿Index test | ﻿Reference standard | ﻿Flow and timing |  | ﻿Patient selection | ﻿Index test | ﻿Reference standard |
| Kuo (2018) | L | L | L | L |  | L | L | L |
| Lee & Kim (2021, sample 1) | L | L | L | L |  | L | L | L |
| Lee & Kim (2021, sample 2) | L | L | L | L |  | L | L | L |
| Lee & Kim (2021, sample 3) | L | L | L | L |  | L | L | L |
| Lee et al. (2024) | L | L | L | L |  | L | L | L |
| Mahmoud et al. (2023, sample 1) | H | H | L | L |  | L | L | L |
| Mahmoud et al. (2023, sample 2) | H | L | L | L |  | L | L | L |
| Mim et al. (2024) | U | L | L | L |  | H | L | H |
| Nawer et al. (2022) | H | L | H | L |  | H | L | U |
| Nawodya &Kumara. (2022) | H | L | U | L |  | L | L | U |
| Oweda et al. (2025) | L | L | L | L |  | L | U | L |
| Pangistu & Azhari (2021,sample 1) | U | L | L | L |  | L | L | L |
| Pangistu & Azhari (2021,sample 2) | U | L | L | L |  | L | L | L |
| Purwandari et al. (2020) | H | L | L | L |  | H | L | L |
| Rahman et al. (2025) | H | L | L | L |  | L | L | L |
| Rho et al. (2016) | H | L | U | L |  | U | L | L |
| Sarkar et al. (2021) | L | L | L | L |  | H | U | L |
| Seo et al. (2020) | L | L | L | L |  | L | L | L |
| Shae & Tsai (2020) | L | L | U | L |  | L | L | L |
| Shin & Dey (2013) | H | L | L | L |  | L | L | L |

QUADAS-2 risk of bias assessment

| Study | ﻿Risk of bias | | | |  | ﻿Applicability concerns | | |
| --- | --- | --- | --- | --- | --- | --- | --- | --- |
|  | ﻿Patient selection | ﻿Index test | ﻿Reference standard | ﻿Flow and timing |  | ﻿Patient selection | ﻿Index test | ﻿Reference standard |
| Singh & Babbar (2018) | H | L | L | L |  | H | L | L |
| Stanimirovic et al. (2024) | L | L | L | L |  | L | L | L |
| Stavropoulos et al. (2023) | L | L | L | L |  | L | L | L |
| Suma et al. (2020, sample 1) | H | L | L | L |  | L | L | L |
| Suma et al. (2020, sample 2) | H | H | L | L |  | H | L | L |
| Sun et al. (2022) | H | L | L | L |  | L | L | L |
| Tsykunov (2020) | L | L | H | L |  | L | L | U |
| Tusher et al. (2022) | H | L | U | L |  | H | L | U |
| Wang et al. (2021) | L | L | L | L |  | L | L | L |
| Wang et al. (2024, sample 1) | L | L | L | L |  | L | L | L |
| Wang et al. (2024, sample 2) | L | L | L | L |  | L | L | L |
| Wan et al. (2025, sample 1) | L | L | L | L |  | U | L | L |
| Wan et al. (2025, sample 2) | L | L | L | L |  | L | L | L |
| Wu & Carette (2020) | L | L | L | L |  | L | L | L |
| Zhang & Yu (2024) | L | L | U | L |  | L | L | L |
| Zhou et al. (2024) | U | L | L | L |  | L | L | L |

*Note.* QUADAS-2 = the revised Quality Assessment of Diagnostic Accuracy Studies. L=low. H=high. U=unclear.

**Methodological quality assessment of included studies using QUADAS-2**


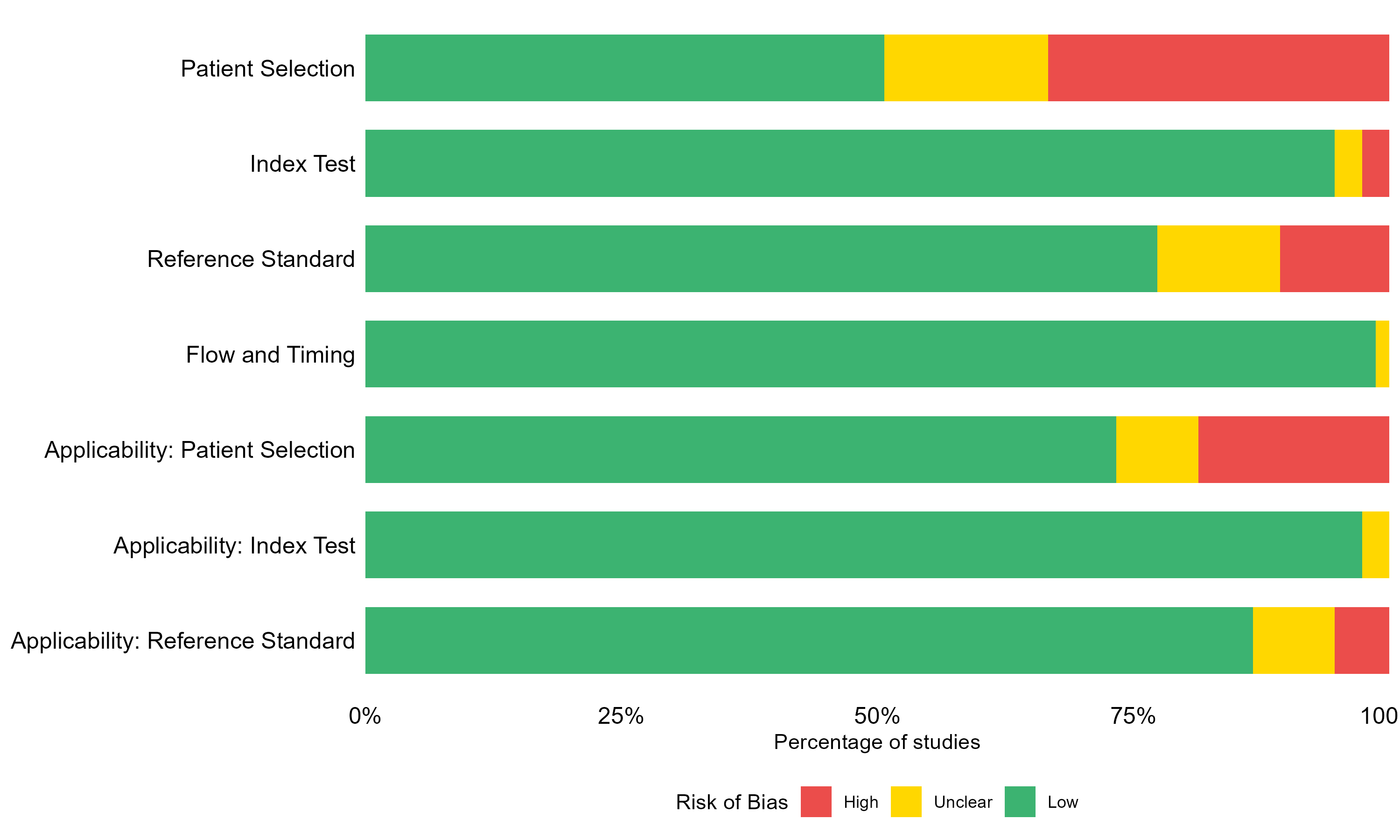


Note: The quality assessment was performed using the QUADAS-2 (Quality Assessment of Diagnostic Accuracy Studies 2) tool for the 64 included studies. The upper four bars represent the "Risk of Bias" in four domains: Patient Selection, Index Test, Reference Standard, and Flow and Timing. The lower three bars represent "Applicability Concerns" regarding Patient Selection, Index Test, and Reference Standard. Green indicates "Low risk," yellow indicates "Unclear risk," and red indicates "High risk."
