# Supplementary material for: Applications of machine learning algorithms to detect digital addiction: a meta-analysis
Source: Front Psychiatry. 2026 Jun 23;17:1789188. doi: 10.3389/fpsyt.2026.1789188 (PMC13338699; doi:10.3389/fpsyt.2026.1789188)
Supplement: Supplemental Table 7 — Full subgroup analysis results. [file Table7.docx]

**Supplemental Material G**

**Table 1:** Subgroup analysis of machine learning predictive accuracy for digital addiction.

| **Moderator** | **Subgroups** | ***k*** | **Pooled Estimate [95% CI]** | ***χ²(p)*** |
| --- | --- | --- | --- | --- |
| Data | Survey | 61 | 0.87 [0.84, 0.89] | 1.97(0.16) |
|  | Physiological | 14 | 0.91 [0.87, 0.94] |  |
| Pub | Unpub | 24 | 0.89 [0.85, 0.92] | 1.36(0.24) |
|  | Pub | 41 | 0.86 [0.83, 0.89] |  |
| Literature Type | Conference Paper | 16 | 0.88 [0.84, 0.92] | 1.06(0.59) |
|  | Unpublished degree theses | 3 | 0.88 [0.80, 0.89] |  |
|  | Peer-reviewed Journal | 24 | 0.85 [0.83, 0.89] |  |
| Gold Standard | Behavioral Data | 15 | 0.81 [0.76, 0.86] | **7.84(0.02^*^)** |
|  | Self-report Questionnaire | 56 | 0.89 [0.86, 0.91] |  |
|  | Interview | 4 | 0.93 [0.84, 0.98] |  |
| Algorithm Category | ML | 56 | 0.88 [0.85, 0.91] | 1.88(0.39) |
|  | EC | 3 | 0.82 [0.67, 0.94] |  |
|  | DL | 16 | 0.85 [0.80, 0.90] |  |
| Specific Algorithm | Support Vector Machine | 15 | 0.89 [0.84, 0.93] | 1.85(0.40) |
|  | Random forest | 11 | 0.85 [0.80, 0.90] |  |
|  | CNN | 3 | 0.85 [0.80, 0.89] |  |
| Validity Validation | Internal Validation | 65 | 0.88 [0.85, 0.90] | 0.98(0.61) |
|  | None | 7 | 0.88 [0.77, 0.96] |  |
|  | External Validation | 3 | 0.80 [0.59, 0.95] |  |
| Validation Methods | Hold-out | 34 | 0.87 [0.84, 0.90] | 1.62(0.66) |
|  | Leave-one-out | 5 | 0.90 [0.86, 0.94] |  |
|  | K-fold cross-validation | 29 | 0.87 [0.83, 0.91] |  |
|  | None | 7 | 0.88 [0.77, 0.96] |  |
| Data Modality | Unimodal | 56 | 0.87 [0.85, 0.90] | 0.02(0.90) |
|  | Multimodal | 19 | 0.87 [0.82, 0.92] |  |
| Data Leakage Prevention | No or Unspecified | 46 | 0.87 [0.84, 0.90] | 0.06(0.80) |
|  | Yes | 29 | 0.88 [0.84, 0.91] |  |
| Addiction Subtype | Smartphone Addiction | 12 | 0.84 [0.78, 0.89] | 4.73(0.32) |
|  | Game Addiction | 24 | 0.85 [0.81, 0.89] |  |
|  | Social Media Addiction | 6 | 0.86 [0.81, 0.91] |  |
|  | Internet Addiction | 27 | 0.90 [0.85, 0.94] |  |
|  | Online Shopping Addiction | 4 | 0.89 [0.84, 0.93] |  |

**Table 2.** Subgroup analysis of machine learning predictive sensitivity for digital addiction.

| **Moderator** | **Subgroups** | ***k*** | **Pooled Estimate [95% CI]** | ***χ²(p)*** |
| --- | --- | --- | --- | --- |
| Data | Survey | 38 | 0.84 [0.79, 0.89] | 3.51(0.06) |
|  | Physiological | 7 | 0.92 [0.86, 0.97] |  |
| Pub | Unpub | 21 | 0.88 [0.82, 0.93] | 0.98(0.32) |
|  | Pub | 24 | 0.84 [0.77, 0.89] |  |
| Literature Type | Conference Paper | 16 | 0.87 [0.79, 0.93] | 0.56(0.76) |
|  | Unpublished degree theses | 3 | 0.88 [0.71, 0.98] |  |
|  | Peer-reviewed Journal | 24 | 0.84 [0.77, 0.89] |  |
| Gold Standard | Behavioral Data | 11 | 0.85 [0.78, 0.90] | 0.08(0.77) |
|  | Self-report Questionnaire | 33 | 0.86 [0.80, 0.91] |  |
| Algorithm Category | ML | 36 | 0.87 [0.83, 0.91] | 1.73(0.19) |
|  | DL | 8 | 0.79 [0.65, 0.90] |  |
| Specific Algorithm | Support Vector Machine | 15 | 0.92 [0.86, 0.96] | **7.86(0.02^*^)** |
|  | Random forest | 11 | 0.82 [0.72, 0.90] |  |
|  | CNN | 3 | 0.68 [0.41, 0.90] |  |
| Validity Validation | Internal Validation | 38 | 0.87 [0.82, 0.91] | 0.66(0.42) |
|  | None | 5 | 0.80 [0.59, 0.95] |  |
| Validation Methods | Hold-out | 20 | 0.83 [0.76, 0.89] | 2.60(0.27) |
|  | K-fold cross-validation | 18 | 0.89 [0.83, 0.94] |  |
|  | None | 5 | 0.80 [0.59, 0.95] |  |
| Data Modality | Unimodal | 37 | 0.85 [0.80, 0.90] | 0.27(0.60) |
|  | Multimodal | 8 | 0.87 [0.79, 0.94] |  |
| Data Leakage Prevention | No or Unspecified | 25 | 0.84 [0.77, 0.90] | 0.89(0.35) |
|  | Yes | 20 | 0.88 [0.82, 0.93] |  |
| Addiction Subtype | Smartphone Addiction | 5 | 0.86 [0.74, 0.95] | 1.57(0.67) |
|  | Game Addiction | 17 | 0.87 [0.82, 0.91] |  |
|  | Internet Addiction | 18 | 0.82 [0.72, 0.91] |  |
|  | Social Media Addiction | 4 | 0.91 [0.79, 0.90] |  |

**Table 3.** Subgroup analysis of machine learning predictive specificity for digital addiction.

| **Moderator** | **Subgroups** | ***k*** | **Pooled Estimate [95% CI]** | ***χ²(p)*** |
| --- | --- | --- | --- | --- |
| Data | Survey | 38 | 0.83 [0.76, 0.89] | **3.03(0.08^+^)** |
|  | Physiological | 7 | 0.90 [0.85, 0.94] |  |
| Pub | Unpub | 21 | 0.84 [0.74, 0.92] | 0.00(0.95) |
|  | Pub | 24 | 0.83 [0.76, 0.90] |  |
| Literature Type | Conference Paper | 16 | 0.84 [0.71, 0.94] | 0.19(0.91) |
|  | Unpublished degree theses | 3 | 0.87 [0.67, 0.98] |  |
|  | Peer-reviewed Journal | 24 | 0.83 [0.76, 0.90] |  |
| Gold Standard | Behavioral Data | 11 | 0.80 [0.67, 0.90] | 0.57(0.45) |
|  | Self-report Questionnaire | 33 | 0.85 [0.78, 0.91] |  |
| Algorithm Category | ML | 36 | 0.83 [0.76, 0.89] | 1.21(0.27) |
|  | DL | 8 | 0.88 [0.81, 0.93] |  |
| Specific Algorithm | Support Vector Machine | 15 | 0.80 [0.64, 0.92] | 2.93(0.23) |
|  | Random forest | 11 | 0.88 [0.83, 0.92] |  |
|  | CNN | 3 | 0.91 [0.85, 0.96] |  |
| Validity Validation | Internal Validation | 38 | 0.84 [0.78, 0.90] | 0.10(0.75) |
|  | None | 5 | 0.86 [0.77, 0.94] |  |
| Validation Methods | Hold-out | 20 | 0.85 [0.78, 0.91] | 0.61(0.74) |
|  | K-fold cross-validation | 18 | 0.81 [0.67, 0.91] |  |
|  | None | 5 | 0.86 [0.77, 0.94] |  |
| Data Modality | Unimodal | 37 | 0.86 [0.80, 0.91] | 1.82(0.18) |
|  | Multimodal | 8 | 0.73 [0.52, 0.90] |  |
| Data Leakage Prevention | No or Unspecified | 25 | 0.86 [0.82, 0.90] | 0.83(0.36) |
|  | Yes | 20 | 0.80 [0.67, 0.91] |  |
| Addiction Subtype | Smartphone Addiction | 5 | 0.79 [0.56, 0.95] | 1.83(0.61) |
|  | Game Addiction | 17 | 0.84 [0.76, 0.92] |  |
|  | Internet Addiction | 18 | 0.84 [0.72, 0.93] |  |
|  | Social Media Addiction | 4 | 0.79 [0.77, 0.82] |  |

**Note.** k = number of studies; CI = confidence interval. *χ²(p)* represents the Q statistic for test of group differences (between-subgroups heterogeneity). ^+^p < .10, ^*^p < .05, ^**^p < .01, ^***^p < .001.
